# Indian research on comorbidities

**DOI:** 10.4103/0019-5545.69240

**Published:** 2010-01

**Authors:** Ashish Srivastava, K. Sreejayan, Anup M. Joseph, P. S. V. N. Sharma

**Affiliations:** Department of Psychiatry, Kasturba Medical College, Manipal, Karnataka - 576 104, India

**Keywords:** Comorbidity, India, last six decades, Psychiatry

## Abstract

The objective of this paper is to provide a review on the psychiatric comorbidity research in India based on the data published in the last six decades. The comorbidity data world over reflects that it is a much more common phenomenon than observed in routine clinical practice. In India, research into this domain of psychiatry has been limited, with comorbidity reported to be as high as 60%. In the few publications in this area, most of the authors have looked into substance related comorbidity. Small numbers of studies have looked into comorbid conditions in child psychiatry, especially mental retardation and very few studies have looked at other comorbidities. The landmarks in the studies in the area of psychiatric comorbidity have been highlighted in this review article.

## INTRODUCTION

The term comorbidity was first introduced by Feinstein in 1970 to denote those cases in which a ‘distinct additional clinical entity’ occurred during the clinical course of a patient having an index disease.[1] Psychiatric comorbidity may be defined as the co-occurrence of two psychiatric disorders in any combinations in the same person. They may occur simultaneously or sequentially. However, it does not necessarily imply that one is caused by the other. These individuals form an important and challenging subset of population associated with poorer outcomes in various clinical domains, including increased risk of relapse, re-hospitalization, life events, suicide and violence, medical comorbidity, homelessness, family discord,[2] economic burden and public healthcare delivery system burden.[3,4] Hence, such a population requires a more proactive outreach throughout the mental health care system. However, the situation seemed to be opposite till last few years. Individuals with co-occurring psychiatric disorders are often perceived to be ‘system misfits’ who have more than one disorder in systems of care that are designed as if everybody had one disorder at any given time.[5] Over the last two decades or so, the multifactorial complexity of comorbidity has been examined in various ways, viz. that individuals actually suffer from multiple disorders, all disorders are offspring of a defective personal, there is one common biological mechanism which leads to more than one disorder, and that disorders are reactions of the individual vulnerabilities to noxious stimuli.[6,7] In this light, there has been increased interest shown by researchers worldwide in the area of comorbidity. Psychiatric comorbidity has been frequently reported in the western literature, the US National comorbidity survey reported 51% of the patients with a diagnosis of major depression had atleast one comorbid anxiety disorder and only 26% had no other comorbid mental disorder.[8]

Similar results have been obtained from the Australian National survey of Mental Health and wellbeing.[9] Comorbidity issues have seemingly been less explored in Indian literature. In the present article, an attempt has been made to review articles relating to comorbidity, which have been researched and published in the Indian Journal of Psychiatry in the last six decades and a few other Indian medical journals and to elaborate on certain important observations.

## SUBSTANCE USE AND COMORBIDITY

To compare substance use in mentally ill and the normal population Dube *et al*.[10] conducted a study in Agra as early as 1962. A total of 29,500 subjects were evaluated out of which 382 patients were “habitual intoxicant users”. The prevalence of substance use was low in the group (0.12%). Details of the pattern of substance use were not assessed in the study. There were 701 mentally ill subjects. Among the substance users the most common substance used was alcohol (62.36%) followed by cannabis (18%). Mentally ill patients were more likely to use multiple substances (30% vs. 16 %). Those using single substance used cannabis more commonly (30%). Both groups used cannabis in the form of bhang more than ganja. Use of opioids was not very common. Nicotine use was not included in the study. In this study, mentally ill were three times more likely to use substance compared to normal people. Patients with psychotic illness were 4.5 times more likely to use substances compared to the normal population. Patients with psychotic illness were three times more likely to use substances compared to those with neurotic illness. Specific life time prevalence was more in patients with bipolar affective illness compared to schizophrenia.

Cannabis has been socially accepted substance in India and has been used in traditional Indian medicine. The psychological consequences of chronic cannabis use have been studied by many Indian researchers. In India it was Dhunjbhoy,[11] who first described what he thought, was “Indian hemp insanity”. Chopra and Chopra[12] concluded that regular cannabis use does not lead to mental derangement or psychosis, whereas Varma[13] and Thacore[14] reported that regular long term use of cannabis can lead to what is described as “Cannabis psychosis” (Varma, 1972) and “Bhang Psychosis” (Thacore, 1973). In a study, Bagadia *et al*.[15] analyzed 20 patients who were taking cannabis regularly; 95% of the subjects were males. It was found that majority of the patients i.e. 17 (85%) had disturbed mental health prior to cannabis consumption. 10 (50%) had schizophrenia, two (10%) were suffering from depression while five (25%) had anxiety disorder and two (10%) had premorbid dissocial personality disorder. The sample was collected from the 2000 new patients, relatives and friends attending the out patient department of psychiatry.

Trivedi *et al*.[16] screened 1000 consecutively presenting patients in a psychiatric hospital and screened them for drug abuse by self report. 16.4% of the patients were drug abusers. In considerations of individual drugs they found that 8.3% of our patients had abused alcohol, 5% cannabis, 2% minor tranquillizer and 0.7% and 0.4% barbiturate and opium respectively. Alcohol was significantly more abused in bipolar subjects and neurotics. Further, cannabis was more abused in schizophrenics and bipolar patients. Although a minor tranquilizer sample showed evidence of higher abuse among patients with schizophrenia and neurotic disorder, yet the difference was not significant.

Kishore *et al*.[17] assessed the lifetime prevalence of comorbidity in 43 patients with substance dependence and the chronology of such comorbidity, using an observational, analytical retrospective study design. The sample was recruited from the de-addiction center of a tertiary hospital at Lucknow. Most common substances used were alcohol (65%) and opioids (56.5%). 60% of the subjects had more than one diagnosis. The commonly co-occurring disorders were mood disorders (35%), sexual dysfunctions (23%), psychotic disorders (11.5%) and anxiety disorders (3.5%). 53.8% with opioid dependence and 30.8% with alcohol dependence had personality disorders. 72.7% of the patients with a diagnosis of Axis II also had an Axis I diagnosis, while 46.9% of those without a personality disorder had Axis I disorders. In five out of nine patients, the mood disorder came primary to the psychoactive substance dependence, four of these patients having dysthymia. In the remaining four patients with mood disorders, substance dependence had preceded the mood disorder in terms of chronology of development. Similarly, in the one patient with schizophreniform disorder, the psychotic episode developed after he had already developed alcohol dependence, the latter thus being the primary diagnosis.

Goswami *et al*.[18] examined the relationship of the courses of substance use and schizophrenic symptomatology in substance abusing “dual-diagnosis” patients with schizophrenia. They concluded that substance use disorder preceded the onset of schizophrenic illness in the majority, and that increase in substance abuse preceded schizophrenic exacerbation in one-third of dual-diagnosis patients. However, overall, they found no evidence that the course of substance use was associated with that of schizophrenia after both disorders were diagnosed.

In a group of 70 patients with schizophrenia, Aich *et al*.[19] found that 54.3% had comorbid substance abuse. Cannabis and nicotine were the commonly abused drugs followed by alcohol. On the sociodemographic profile the schizophrenic patients who abused drugs did not differ from the ones who did not. It was seen that substance abusing schizophrenics were clustered in the positive syndrome group and that non-substance abuse schizophrenics clustered in the negative and mixed syndrome group.

In another study on 30 subjects with alcohol dependence, by Vohra *et al*.[20] 23 (76.6%), patients were found to have comorbid psychiatric disorder. The axis - I comorbidty was found in 76.6% patients. Axis - II comorbidity was seen in 40% of the sample. Cluster B personality disorders accounted for the maximum (58.3%) axis - II comorbidity. Major depressive disorder was diagnosed 52.1% of patients.

In a study examining the comorbidity in alcohol dependence by Singh H.N. *et al*.[21] the prevalence of psychiatric disorders was found to be 92% compared to 12% in controls. The most common disorders were depression (26%), dissocial personality disorder (21%) and phobias (16%).

## MOOD DISORDER AND COMORBIDITY

Hundred subjects who met the criteria for current manic episode were recruited from a psychiatric hospital and were evaluated for substance abuse by Suresh *et al*.[22] 50% of the subjects had comorbid substance abuse. The commonest drug abused was alcohol with 23.8% at abuse level and 14.9% at dependence level. Prevalence of cannabis used disorders was 26.7%, sedative-hypnotics 4%, cocaine 3%, nicotine 10% and polysubstance abuse 3%. Substance abuse was the single most consistent factor found to be associated with poor outcome of mania.

Mendhekar and Mehta[23] published a case report documenting the presence of multiple paraphilias during a manic episode in a 60-year-old male subject. The authors suggest that in mania, lack of proper sexual outlet and social boycott might be the contributory factors for exhibiting abnormal sexual behavior. Gupta and Basu[24] reported a case of recurrent mania secondary to alcohol intake and with a similar family history. The report highlighted the potential role of alcohol as a mania inducing agent.

## SCHIZOPHRENIA AND DEPRESSION

S.S. Raju[25] studied the comorbidity of depressive disorders in schizophrenia. Of the 529 patients with schizophrenia recruited into the study, 34% were found to have depressive symptoms. After eight weeks of treatment with neuroleptics, in 47% of the patients the depressive symptoms abated. These patients were followed up for six to 48 months; 10% of the subjects developed major depressive disorder in presence of residual symptoms; 25% of patients with residual symptoms developed depressive symptoms not amounting to a syndrome; 3% developed schizo-affective disorders; 2% of the patients in whom the schizophrenic symptoms had remitted, developed major depressive disorder and 53% of the subjects who developed depressive symptoms on follow-up had no such symptom at the onset of schizophrenic illness.

## MENTAL RETARDATION AND COMORBIDITY

A study conducted in the child psychiatry unit of a tertiary psychiatric hospital by Khess R.J. *et al*.[26] evaluated the comorbidity in children with mental retardation-57% of the subjects had some psychiatric comorbidity. The comorbidities found were mood disorder (8%), hyperkinetic disorder (14%), autism (11%), psychosis (11%), conduct disorder (2%) enuresis (2%) and unspecified emotional and behavioral disorder (26 %). Mood disorders were found to be common in children with mild level of mental retardation. It was observed that patients with a psychiatric disorder had a milder level of retardation compared to patients with a medical illness. The psychiatric illness and medical illness did not co exist frequently.

In a cross sectional study, Bhattacharyya *et al*.[27] found that contrary to the conventional belief, individuals diagnosed with Down syndrome had higher frequency of behavioral abnormalities such as impulsivity and stereotypies compared to the normal population.

Greydanus and Pratt[28] in a review of syndromes associated with mental retardation mentions the externalizing behavior problems seen in these individuals. Adolescents with severe Mental retardation often exhibit self stimulatory and self injurious behavior patterns apart from mild to severe tantrums. The authors opine that the caregivers should make efforts to minimize the youth’s capacity for self harm. They further state that youths with mild mental retardation constitute the highest risk group for engaging in high risk sexual behavior and becoming victims of physical, sexual and mental abuse. Depressive disorders in adolescents with mental retardation confer an increased risk for illness and interpersonal difficulties apart from an increased risk for substance abuse and suicidal behavior. The authors underscore the risks of not detecting and treating depression in this group of youth.

## ATTENTION DEFICIT HYPERACTIVITY DISORDER AND SPECIFIC LEARNING DISORDERS

Karande *et al*.[29] documented the clinical profile and academic history of children with specific learning disability and co-occurring ADHD. They found that all the children in their study had poor academic performance and about 40% of the sample had developed aggressive or withdrawn behavior. The authors also found no significant gender differences in the clinical profile. Karande and Bhosrekar[30] evaluated the impact of co-occurring attention deficit hyperactivity disorder (ADHD) on the health-related quality of life (HRQOL) of children with newly diagnosed specific learning disability (SpLD). A little more than a quarter of the 150 children enrolled in the study had co-occuring ADHD. The study indicated that co-occurring ADHD adversely affected the HRQOL of children with SpLD and highlighted the importance of treating co-occurring ADHD effectively to improve the psychosocial health of such children. In an editorial, Susan Crawford felt that a lack of awareness was a major reason for disorders such as specific learning disabilities (SpLD) and attention-deficit hyperactivity disorder (ADHD) in children to go unidentified in India.[31]

## PERVASIVE DEVELOPMENTAL DISORDER AND COMORBIDITY

Girimaji SR *et al*.[32] at NIMHANS, Bangalore studied comorbid psychiatric disorders in children with pervasive developmental disorders. In the sample of 50 children, comorbidity was evident in 46%. The common comorbid conditions observed were attention deficit hyperactivity disorder, anxiety disorder including obsessive compulsive disorder, bipolar affective disorder and circadian distur bance of sleep. There was a significant group of cases with subsyndromal comorbid diagnoses.

## CONCLUSION

Evaluation and diagnosis of comorbid disorder is of paramount importance in order to modify treatment schedules and improve patient outcomes. In the present times, comorbid diagnosis should be an expectation and not an exception. Studies have consistently shown in the past that appropriate psychopharmacology of a known psychiatric disorder ensures better outcome for both the index mental illness and comorbid diagnosis with special reference to substance use. At the same time we now have some literature bringing out the fact that the treatment of both index and comorbid conditions is necessary, for example, integrated mental health and substance abuse treatment is the best treatment practice. Treatment of patients with comorbid diagnosis should be individually matched and based on assessment of diagnoses, level of disability, stage of change, treatment, rehabilitative goals or strengths, and level of care. Treatment should also be stage specific including individual and family interventions, community reinforcement and contingency management. Each of these areas need further research in relation to specific comorbidity combinations. It can be seen that the research so far has started to address some of the above issues but many of them remain unexplored.
